# Complete Genome Sequences of Nine Streptococcus pneumoniae Serotype 3 Clonal Complex 180 Strains

**DOI:** 10.1128/mra.00275-22

**Published:** 2022-05-31

**Authors:** Smitha Shambhu, Eleonora Cella, Mohammad Jubair, Taj Azarian

**Affiliations:** a Burnett School of Biomedical Sciences, University of Central Florida, Orlando, Florida, USA; University of Maryland School of Medicine

## Abstract

We announce the complete genomes of nine Streptococcus pneumoniae strains belonging to serotype 3 clonal complex 180 (CC180). The genomes consist of a single circularized contig with an average length of 2.033 Mbp. Pangenome analysis identified 1,762 core genes and 412 accessory genes. These genomes are the basis for future population genomic studies.

## ANNOUNCEMENT

Streptococcus pneumoniae is a commensal bacterium found in the human nasopharynx that can cause the invasive diseases of pneumonia, otitis media, meningitis, and bacteremia. Of the 100 identified serotypes, serotype 3 is highly invasive and is associated with a high risk of death (1). Nine serotype 3 strains were obtained from a culture collection of samples collected during a carriage study of Massachusetts children that was conducted between 2000 and 2014 (2, 3). Previous population genomic analysis of draft assemblies identified that they belonged to two divergent clades of clonal complex 180 (CC180), termed clade Iα and clade II (3, 4). Clade II is of particular interest due to its increased prevalence after the introduction of the 13-valent pneumococcal conjugate vaccine (PCV13).

Strains were grown overnight at 37°C in 5% CO_2_ in Bacto Todd-Hewitt broth (BD, Heidelberg, Germany) containing 0.5% yeast extract (BD). Genomic DNA (gDNA) was extracted and purified using the Qiagen DNeasy blood and tissue kit according to the manufacturer’s instructions. Enzyme lysis buffer for Gram-positive bacteria was prepared according to instructions with the addition of 100 mg/mL lysozyme. An overnight 5-mL culture was centrifuged at 5,000 × *g* for 10 min, and 360 μL of the lysis buffer and lysozyme mixture was added to each cell pellet and incubated at 37°C for 1 h. The quality and concentration of gDNA were assessed using the Agilent 4200 TapeStation system and a Qubit 4 fluorometer.

Using an Oxford Nanopore Technologies (ONT) MinION system, a ligation sequencing kit, and an R9.4.1 flow cell, we produced an average of 330 Mbp of sequencing data for each strain. We performed base calling using Guppy v0.5.1 with FAST mode and adapter trimming with Porechop v0.2 (5). Reads were filtered with Filtlong v0.2.0 (https://github.com/rrwick/Filtlong) using the settings –min_length 1000 and –target_bases 84,000,000. The final long-read data set for the nine strains had mean read lengths that ranged from 4,495 to 11,490 bp (minimum *N*_50_, 6,120 bp) and a mean read quality score of 10.0. Data quality was assessed using NanoPlot v1.0.0 (6). Hybrid assemblies were generated from ONT data and previously published Illumina short-read data (BioProject accession number PRJNA437292; detailed accession numbers are in Table 1) using Unicycler v0.4.8, which resolved a single circularized unitig (7). Three samples required an alternative approach using Trycycler v0.5.1 to obtain circularized assemblies (8). With both approaches, assemblies were error corrected (i.e., polished) using Illumina short reads and reordered to begin at the start position of *dnaA*. Default parameters were used for all software unless otherwise specified. The final error-corrected assemblies have a total average length of 2,033,799 bp and a GC content of 39.7%. The genomes were annotated using the NCBI Prokaryotic Genome Annotation Pipeline (PGAP) (9). The genomes consist of, on average, 2,091 total annotated genes, 2,018 protein-coding sequences, 58 tRNAs, four 5S rRNAs, four 16S rRNAs, and four 23S rRNAs. Pangenome analysis was performed using Roary v3.13.0, which identified 1,762 core genes shared by all nine genomes and 412 accessory genes (10).

**TABLE 1:** Data on the nine Streptococcus pneumoniae serotype 3 CC180 strains

Taxon	BioSample accession no.	SRA accession no.		ONT sequencing data				Hybrid assembly GenBank accession no.	Genome size (bp)	GC content (%)	No. of coding sequences
		Illumina sequencing reads	ONT sequencing reads	Total no. of bases	*N*_50_ (bp)	Mean read quality score	Mean read length (bp)				
PT8465	SAMN08647902	SRX3774795	SRR17486872	110,005,738	8,137	10.7	8,324.3	CP090888	2,003,561	39.8	2,087
LE4448	SAMN08647548	SRX3775148	SRR17486877	110,000,850	6,994	10.1	6,455.4	CP090883	2,003,818	39.8	2,068
CH2439	SAMN08647378	SRX3775100	SRR17486871	110,000,620	6,120	10.0	4,495.9	CP090889	2,003,571	39.8	2,067
CH2241	SAMN08647361	SRX3775363	SRR17486878	110,005,162	8,353	10.4	8,292.3	CP090882	2,003,723	39.8	2,114
NP7536	SAMN08647838	SRX3774973	SRR17486873	110,003,273	10,791	10.8	10,796.3	CP090887	2,046,177	39.8	2,115
ND6401	SAMN08647706	SRX3775069	SRR17486875	110,007,739	11,320	10.6	11,490.3	CP090885	2,057,101	39.7	2,120
MD5403	SAMN08647626	SRX3775317	SRR17486876	110,000,583	8,689	10.6	8,589.1	CP090884	2,061,648	39.7	2,068
NP7513	SAMN08647831	SRX3774783	SRR17486874	110,002,691	11,243	10.9	11,248.9	CP090886	2,062,504	39.7	2,111
BR1268	SAMN08647280	SRX3774733	SRR17486879	110,003,733	9,599	10.5	9,777.2	CP090881	2,062,088	39.7	2,067

### Data availability.

Whole-genome shotgun projects have been deposited in GenBank under the accession numbers CP090888, CP090883, CP090889, CP090882, CP090887, CP090885, CP090884, CP090886, and CP090881. The versions described in this paper are versions CP090881.1 to CP090889.1. The raw sequence reads are available under BioProject accession number PRJNA437292, with the BioSample accession numbers SAMN08647902, SAMN08647548, SAMN08647378, SAMN08647361, SAMN08647838, SAMN08647706, SAMN08647626, SAMN08647831, and SAMN08647280. An extended version of Table 1 with additional metadata is available at https://doi.org/10.6084/m9.figshare.19654020.v1.
